# The Emerging Role of Neural Cell-Derived Exosomes in Intercellular Communication in Health and Neurodegenerative Diseases

**DOI:** 10.3389/fnins.2021.738442

**Published:** 2021-08-31

**Authors:** Luyao Huo, Xinzhe Du, Xinrong Li, Sha Liu, Yong Xu

**Affiliations:** ^1^Department of Psychiatry, First Hospital/First Clinical Medical College of Shanxi Medical University, Taiyuan, China; ^2^Shanxi Key Laboratory of Artificial Intelligence Assisted Diagnosis and Treatment for Mental Disorder, First Hospital of Shanxi Medical University, Taiyuan, China; ^3^Department of Mental Health, Shanxi Medical University, Taiyuan, China

**Keywords:** exosomes, extracellular vesicles, intercellular communication, neurons, glial cells, health, neurodegenerative diseases

## Abstract

Intercellular communication in the central nervous system (CNS) is essential for brain growth, development, and homeostasis maintenance and, when dysfunctional, is involved in the occurrence and development of neurodegenerative diseases. Increasing evidence indicates that extracellular vesicles, especially exosomes, are critical mediators of intercellular signal transduction. Under physiological and pathological conditions, neural cells secret exosomes with the influence of many factors. These exosomes can carry specific proteins, lipids, nucleic acids, and other bioactive substances to the recipient cells to regulate their function. Depending on the CNS environment, as well as the origin and physiological or pathological status of parental cells, exosomes can mediate a variety of different effects, including synaptic plasticity, nutritional metabolic support, nerve regeneration, inflammatory response, anti-stress effect, cellular waste disposal, and the propagation of toxic components, playing an important role in health and neurodegenerative diseases. This review will discuss the possible roles of exosomes in CNS intercellular communication in both physiologic and neurodegenerative conditions.

## Introduction

Normal functions of the central nervous system (CNS) rely critically on the exchange and integration of information among neural cells, including neurons, microglia, astrocytes, and oligodendrocytes. Communication of neural cells is widely involved in brain development and homeostasis, as well as the occurrence of nervous system diseases such as neurodegenerative diseases (Stogsdill and Eroglu, 2017; Afridi et al., 2020). In general, cell-to-cell communication can be mediated by direct intercellular contact or the release of bioactive factors (Raposo and Stoorvogel, 2013). In recent years, extracellular vesicles (EVs), especially exosomes, have attracted growing attention as a new intercellular communication mechanism.

**FIGURE 1:**
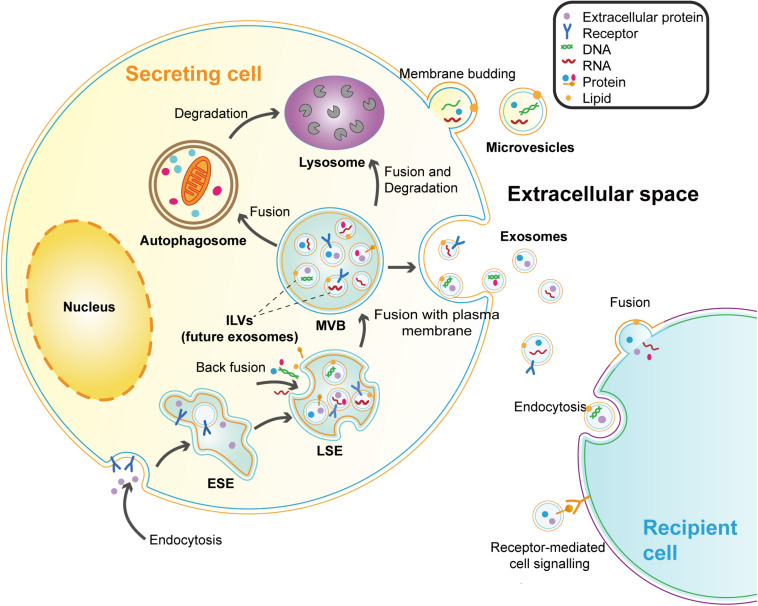
Biogenesis and secretion of exosomes and microvesicles. Microvesicles are directly budding from the plasma membrane (PM). Exosomes are vesicles originating from the endosomal system. The early-sorting endosomes (ESEs) are formed by endocytosis and PM invagination. During this process, some extracellular constituents can enter the ESEs along with cell-surface proteins. Then the ESEs mature into late-sorting endosomes (LSEs). Following the specific cargos (proteins, lipids, or nucleic acids) accumulating at the endosomal membrane, the limiting membrane of LSEs further bud inward to generate the intraluminal vesicles (ILVs) which are future exosomes. LSEs develop into MVBs with the collection of ILVs. MVBs can be transported to the PM and release exosomes by exocytosis. MVBs can also participate in the degradation pathway by fusing with autophagosomes or lysosomes. Because of the double invagination processes, the exosomal membrane maintains the same topological orientation as the PM of cells. Exosomes can be taken up by recipient cells in different ways, including endocytosis, receptor-ligand interaction, and fusion with the recipient cell membrane.

Extracellular vesicles are membrane vesicles secreted by cells into the extracellular matrix and have been observed in most types of cells (Bang and Thum, 2012). According to biological origin and size, EVs can be divided into three categories: apoptotic bodies (500–2000 nm), microvesicles (200–2000 nm), and exosomes (40–200 nm) (Shao et al., 2018). Apoptotic bodies are specific vesicles budding from the plasma membrane (PM) during the process of apoptosis (Todorova et al., 2017). Microvesicles (MVs) refer to the vesicles that shed directly from the PM of live cells (Shao et al., 2018; Russell et al., 2019). While exosomes are derived from intraluminal vesicles (ILVs) formed in the multivesicular bodies (MVBs) that are released into the extracellular milieu by the fusion of MVBs and PM (Russell et al., 2019). Exosomes seem to be the most characteristic EVs and are widely detected in biofluids, such as plasma, urine, saliva, cerebrospinal fluid, and breast milk (Raposo and Stoorvogel, 2013). The biogenesis process and major features of exosomes are presented below (Figure 1 and Table 1).

**TABLE 1:** Major features of exosomes.

Feature	Exosomes	References
Size	40–200 nm	Shao et al., 2018
Markers	ESCRT mechanism components (Alix, TSG101), tetraspanins (CD9, CD63, and CD81), heat shock proteins (Hsp70 and Hsp90), Rab GTPases, flotillin	Kalluri and LeBleu, 2020
Cargos	Proteins; lipids; DNA, mRNA, microRNA, and other non-coding RNAs	van Niel et al., 2018
Mechanism of release	**ESCRT-dependent pathways**	
	ESCRT-0, I, II, and III: MVBs formation, vesicle budding, and protein cargo sorting	Zhang et al., 2019
	**ESCRT-independent pathways**	
	Ceramides: RNA sorting in mammalian cells, spontaneous negative curvature of the endosomal membrane and inducing the budding of ILVs	Trajkovic et al., 2008; Kosaka et al., 2010
	Tetraspanins such as CD9, CD63, CD82: the sorting of cargos to ILVs	Chairoungdua et al., 2010; van Niel et al., 2011
	Rab family proteins such as Rab27A, Rab27B, and Rab35: inducing the transfer of MVBs to the cell surface	Hsu et al., 2010; Ostrowski et al., 2010
	Cytoskeleton, molecular switches (small GTPases), membrane fusion associated molecules (SNAREs, tethering factors): the docking and fusion process between MVBs and PM	Raposo and Stoorvogel, 2013
Mechanism of targeting recipient cells	Endocytosis (micropinocytosis, endocytosis mediated by clathrin, caveolin, or lipid rafts, phagocytosis of specialized cells such as macrophages)	van Niel et al., 2018
	Fusing directly with the PM of the recipient cells	van Niel et al., 2018
	Receptor-ligand interactions	Mulcahy et al., 2014
Physiological effect	Removing unnecessary components from cells: Modulating the expression and function of recipient cells by transferring bioactive molecules	Kalluri and LeBleu, 2020

*ESCRT, endosomal sorting complex required for transport; SNAREs, soluble N-ethyl maleimide-sensitive factor attachment protein receptors.*

Increasing evidence showed that neural cell-derived exosomes are vital signal carriers of the CNS. By carrying bioactive molecules and mediating signal transmission of adjacent or distant cells, exosomes may play a crucial role in intercellular communication and neurodegenerative diseases. Besides, neural exosomes can cross the blood-brain barrier (BBB), implicating that exosomes may be a potential window into brain physiological and pathological activities. Exosomes can be used as molecular markers as well as carriers of drugs and bioactive molecules, and have good potential in the diagnosis and therapy of neurodegenerative diseases (Mustapic et al., 2017). In this review, we aim to clarify the role of neural cell-derived exosomes in physiological conditions and diseases, predominantly in neurodegenerative diseases. We first introduce the general characteristics of exosomes from neural cells, including neurons, glial cells, and neural stem/progenitor cells (NSCs). Then, we focus on the function of neural exosomes in CNS intercellular communication both in health and neurodegenerative diseases. Finally, we discuss the potential of exosomes in the therapy of neurodegenerative diseases.

## Characteristics of Exosomes in the CNS

Besides the general features, exosomes released by neural cells also exhibit some specific characteristics depending on their cell origins. A substantial body of researches have explored the inducible factors and secretion mechanism of exosomes in CNS (Table 2).

**TABLE 2:** Characteristics of exosomes secreted by neural cells.

Cell types	Specific markers	Secretion regulation		
		Stimulus	Mechanism	References
Neurons	L1CAM, GluR2/3 (Fauré et al., 2006)	K^+^	Synaptic activity	Fauré et al., 2006
		Calcium	Synaptic activity	Lachenal et al., 2011
		Glutamatergic synapse	Activating NMDA and AMPA, increasing intracellular calcium	Lachenal et al., 2011
		bFGF	Increasing the MVB-PM fusion of cultured hippocampal neurons	Kumar et al., 2020
Microglia	Aminopeptidase CD13, Lactate transporter MCT-1 (Potolicchio et al., 2005)	LPS, IL-4	Polarization of microglia	Verderio et al., 2012; Cunha et al., 2016; Yang Y. et al., 2018; Tian et al., 2019
		ATP	Activating P2X7R	Bianco et al., 2005, 2009; Takenouchi et al., 2015
		Serotonin (5-HT)	5-HT_2a,b_- Gαq proteins- PLC; 5-HT_4_- Gαs proteins- AC-cAMP- GEF1/2- Rap1-PLC; PLC- calcium influx- the fusion of MVBs and PM	Glebov et al., 2015
		Wnt3a	GSK3-independent mechanism	Hooper et al., 2012
Astrocytes	GFAP, GLAST, GLUL (Goetzl et al., 2016)	ATP	Binding to P2X7R and activating the sphingomyelinase	Bianco et al., 2009
		IL-1β, IL-10 (EVs)	–	Datta Chaudhuri et al., 2020
		PrP	PrP- CAV1 internalization- inhibiting the formation of ATG12- ATG5 complex- protect the MVBs from degradation	Dias et al., 2016
		HIV Tat	–	Wang et al., 2012; Yang L. et al., 2018
		Amyloid peptide	Ceramide produced by nSMase2: the formation and secretion of exosomes	Wang et al., 2012
		Aβ (inhibit secretion)	Increasing the JNK phosphorylation and activating the JNK signal pathway	Abdullah et al., 2016
		Mutant huntingtin (inhibit secretion)	Inhibiting the transcription of αB-crystallin	Hong et al., 2017
Oligodendrocytes	PLP, CNP MBP, MOG (Krämer-Albers et al., 2007)	Glutamatergic synapse	Activating NMDA and AMPA, increasing intracellular calcium	Frühbeis et al., 2013
		Rab35	Facilitating the docking of endosomes with oligodendrocyte membrane	Hsu et al., 2010
Neural stem cells	–	Pro- and anti-inflammatory cytokines	–	Cossetti et al., 2014; Zhang et al., 2020

### Neuron-Derived Exosomes

The neuron is the basic structural and functional unit of the CNS. The main function of neurons is to receive, integrate, and transport information to maintain the proper activity of the CNS. The release of exosomes has been demonstrated both in developing neurons and differentiated mature neurons. These neuron-derived exosomes (NDEs) contain some neuron-specific markers, including L1 cell adhesion molecule (L1CAM) and the GluR2/3 subunits of glutamate receptors (Fauré et al., 2006; Lachenal et al., 2011). Exosomes stem from MVBs, which can be transported in neurons and release exosomes by fusing with the PM of the soma, the dendrites, the axons, or the presynaptic membrane (Janas et al., 2016).

The secretion of exosomes is regulated by synaptic activity (Table 2). Fauré et al. (2006) proved that a high concentration of K^+^ stimulation could promote the secretion of NDEs, suggesting that exosome secretion can be modulated by depolarization. The team then confirmed that the release of exosomes from well-differentiated neurons was influenced by calcium influx and glutamatergic synaptic excitability (Lachenal et al., 2011). Interestingly, recent research shows that high-frequency electrical stimulation (HFS) can evoke a fast and immediate increase of intracellular calcium, while the fusion of MVBs and the PM has a time lag after HFS. The result suggests that, unlike synaptic vesicles, the fusion of MVBs and the PM is not directly coupled to the calcium influx and may involve a complex signal network. This study also found that the basic fibroblast growth factor (bFGF), a family of pleiotropic growth and differentiation factors, could increase the MVB-PM fusion and the release of exosomes in cultured hippocampal neurons (Kumar et al., 2020).

The binding of exosomes to neurons seems to be of specificity. Exosomes released by cortical neurons upon synaptic activation are reported to be selectively absorbed by neurons but not by glial cells, suggesting that NDEs can specifically interact with other neurons and this may be a novel aspect of neuronal communication (Chivet et al., 2014). Strikingly, it has been confirmed that some NDEs internalized into neurons can be re-secreted together with endogenous exosomes of the recipient neurons and this might result in a longer-distance action and increase the potential of exosomes for cell communication and widespread physiological or pathological signals (Polanco et al., 2018).

### Microglia-Derived Exosomes

Microglia are the first line of defense against pathogens and brain injury in the CNS. By screening the brain parenchyma, microglia can quickly detect the signals of pathogen invasion, tissue damage, or internal environment disorders, and further participate in immune regulation, tissue repair, and remodeling to maintain the homeostasis of the CNS (Ginhoux et al., 2013; Caruso Bavisotto et al., 2019). There is increasing evidence in support of the presence of microglia-derived EVs, including exosomes and MVs (Bianco et al., 2005; Potolicchio et al., 2005). Analysis of protein content showed that microglia-derived exosomes (MDEs) contain several unique markers, including the aminopeptidase CD13 and the lactate transporter MCT-1 (Potolicchio et al., 2005).

Some studies have explored the stimulus and downstream signaling pathways that induce exosome shedding from microglia (Table 2). The secretion of MDEs is closely related to infection and neuroinflammation. Lipopolysaccharide (LPS) is a component of the cell wall of Gram-negative bacteria. Exposure to LPS or neuroinflammatory factors such as IL-4 has been identified to significantly increase the release of MDEs and modulate their biogenesis and composition, thereby regulating the neuroinflammatory (Cunha et al., 2016; Tian et al., 2019). Moreover, extracellular adenosine triphosphate (ATP) was showed to induce the secretion of MDEs and MVs by activating P2X7 receptors (P2X7R) (Bianco et al., 2005; Takenouchi et al., 2015). Another study has shown that ATP can further modify the protein composition of MDEs, promoting the sorting of proteins associated with the autophagy-lysosomal pathway, cellular metabolism, cell adhesion, and extracellular matrix organization, thereby influencing the function of MDEs (Drago et al., 2017). Neurotransmitter was also involved in the release of MDEs. Serotonin (5-HT) secreted by neurons was proved to stimulate the release of MDEs by binding to the 5-HT_2a,b_, and 5-HT_4_ receptors in microglia. The 5-HT_4_ could transmit signals to adenylate cyclase (AC) *via* Gαs proteins to regulate the generation of cAMP then the cAMP activates GEF1/2 to transmit signals to phospholipase C (PLC) *via* Rap1. While 5-HT_2a,b_ transmit signals to PLC *via* Gαq proteins. PLC can further mediate the increase of cytosolic calcium, promoting the fusion of MVBs and cell membranes to release exosomes (Glebov et al., 2015). Additionally, Wnt3a was capable of stimulating the release of exosomes from microglia through a GSK3-independent mechanism without altering the release of primary cortical neuron exosomes. Wnt3a-induced MDEs contain a variety of proteins related to cellular structure, metabolism, protein synthesis, and decomposition (Hooper et al., 2012).

### Astrocyte-Derived Exosomes

Astrocytes are the most abundant glial cells in the CNS and are essential for brain homeostasis maintenance (Colombo and Farina, 2016). Under physiological conditions, astrocytes play a variety of complex and important roles in the CNS, including providing metabolites and growth factors for neurons, promoting synapse formation, modulating synaptic transmission by removing or recycling neurotransmitters, and contributing to the maintenance of the BBB (Freeman and Rowitch, 2013). Moreover, astrocytes are also important regulatory cells of brain immune function and play an important part in controlling the transport and activation of immune cells (Colombo and Farina, 2016). Some studies provided preliminary evidence for the secretion of exosomes from cultured astrocytes (Taylor et al., 2007; Guescini et al., 2010). Some astrocyte-specific proteins including glial fibrillary acidic protein (GFAP), glutamine aspartate transporter (GLAST), and glutamine synthetase (GLUL) have been found in astrocyte-derived exosomes (ADEs) (Goetzl et al., 2016).

Similar to microglia, the release of ADEs is also influenced by the trophic stimulus, inflammatory stimulus, and other microenvironmental factors (Table 2). A recent study showed that ATP could induce astrocytes to release IL-1β in exosomes and MVs by binding to P2X7R and rapidly activating the sphingomyelinase (Bianco et al., 2009). In another recent study, both pro-inflammatory factors (IL-1β) and anti-inflammatory factors (IL-10) have been suggested to induce the release of EVs in astrocytes. Notably, the proteomic analysis found that EVs exposed to IL-10 or ATP were rich in proteins associated with axonal growth, dendrite branching, and synapse formation. By contrast, IL-1β-induced EVs contain proteins involved in regulating peripheral immune response and promoting the recruitment of immune cells to CNS, which potentially mediate the transmission of inflammatory signals (Datta Chaudhuri et al., 2020). This result suggests that these stimulating factors could induce the release of astrocyte EVs, as well as participate in the modification of the EVs cargos. Nonetheless, the specific signaling pathways still need to be further studied.

Stimulating with cytotoxic proteins such as HIV trans-activating regulatory protein (Tat) and amyloid peptide can promote the secretion of ADEs, thus mediating cell damage of the CNS (Wang et al., 2012; Yang L. et al., 2018). In the case of HIV infection, Tat-induced astrocytes have been observed to release exosomal miR-9 which was subsequently internalized by microglia and mediated microglial migration (Yang L. et al., 2018). It was reported that the amyloid peptide could induce astrocytes to secrete exosomes rich in ceramide and prostate apoptosis response 4 (PAR-4) and promote cell apoptosis. And the ceramide produced by neutral sphingomyelinase 2 (nSMase2) displays a crucial role in the formation and secretion of ADEs (Wang et al., 2012). Additionally, prion proteins (PrP) localized on the cell membrane were proved to be a positive regulator of exosome secretion and promote the release of ADEs by inhibiting the degradation pathway of MVBs. This process may depend on the role of PrP in facilitating the internalization of CAV1 and subsequently inhibiting the formation of the autophagy-related 12 (ATG12)-ATG5 complex, thus impairing the autophagy progression and protecting the MVBs from fusion with autophagosomes and their transport to lysosomes for degradation, thus promoting the release of ADEs (Dias et al., 2016). Nevertheless, some researches found that some cytotoxic proteins can reduce the release of ADEs, thereby inhibiting the neuroprotective effects of astrocytes (Abdullah et al., 2016; Hong et al., 2017). Beta-amyloid (Aβ) appears to suppress the secretion of ADEs by increasing the JNK phosphorylation and activating the JNK signal pathway. Aβ in the brain may impair exosome-mediated Aβ clearance through this mechanism (Abdullah et al., 2016). Moreover, αB-crystallin, a small heat shock protein that is abundant in astrocytes (Hong et al., 2017), has been shown to mediate exosomal secretion in cultured human retinal pigment epithelial cells (Gangalum et al., 2016). Mutant huntingtin (mHtt) has been found to inhibit the transcription of αB-crystallin and further reduce the secretion of ADEs, which potentially play a neuronal protective role in the brain of Huntington’s disease (Hong et al., 2017).

### Oligodendrocyte-Derived Exosomes

Oligodendrocytes are responsible for myelin formation, maintenance, and nutritional support for axons (Reiter and Bongarzone, 2020). Oligodendrocytes have been demonstrated to secrete high levels of exosome-like vesicles (Krämer-Albers et al., 2007; Frühbeis et al., 2013). Proteomic analysis showed that besides the typical exosomal markers such as Alix and Tsg101, oligodendrocyte-derived exosomes (ODEs) also contained some specific components of myelin, including myelin proteolipid protein (PLP), 2′3′-cyclic-nucleotide-phosphodiesterase (CNP), myelin basic protein (MBP), myelin oligodendrocyte glycoprotein (MOG), and typical myelin lipids (Krämer-Albers et al., 2007). Under electron microscopy, PLP-positive MVBs were observed present in the cytoplasm of oligodendrocytes near axons and could fuse with the PM and release exosomes to the extracellular matrix around axons, which may participate in the oligodendrocyte-axon interaction (Frühbeis et al., 2013).

The release of ODEs is closely relevant to the activity of neurons (Table 2). The glutamate released by neuron depolarization can mediate calcium influx by activating ionotropic glutamate receptors including N-methyl-D-aspartate (NMDA) and α-amino-3-hydroxy-5-methyl-4-isoxazolepropionic acid (AMPA) on the surface of oligodendrocytes, which leads to increased intracellular calcium levels and triggers exosome secretion (Frühbeis et al., 2013). In addition, the Rab family guanosine triphosphatases (GTPases) serve as master regulators of membrane transport. Screening of a Rab GTPase-activating protein library identified that Rab35 and its activating proteins TBC1D10A-C can modulate the release of ODEs. Rab35 was detected at the surface of oligodendrocytes and can promote the recruitment of endosomes to the PM, while reducing the activity of Rab35 would lead to intracellular accumulation of endosomes and inhibit exosomal secretion, suggesting that Rab35 potentially promote exosome secretion by facilitating the docking of endosomes with oligodendrocyte membrane (Hsu et al., 2010).

### Neural Stem Cell-Derived Exosomes

Neural stem/progenitor cells are self-renewal multipotent cells and are capable of generating neurons, astrocytes, and oligodendrocytes during the development of the CNS (McKay, 1997). In adults, there are still a small number of NSCs in the sub-ventricular zone (SVZ) and the subgranular zone of the hippocampal dentate gyrus (Ma et al., 2009). As a special type of stem cell that only exist in the CNS, NSCs can differentiate into three types of neural cells to maintain and repair damaged brain tissue, thus playing an important role in the treatment of nervous system diseases (Vogel et al., 2018). NSCs can also secrete exosomes, which carry exosomal markers such as PDCD6IP (Alix), TSG101, CD63, and CD9 (Vogel et al., 2018). Through the next-generation sequencing technology, Stevanato et al. (2016) identified a set of specific miRNAs in NSC-derived exosomes. Among these, miR-1246, miR-4488, miR-4508, miR-4492, and miR-4516 were highly enriched exosomal miRNAs and further participated in the gene regulation of target cells (Stevanato et al., 2016). There are few studies on the stimulus and downstream pathways that induce exosomal secretion from NSCs. Cossetti et al. (2014) have shown that the stimulation of pro- and anti-inflammatory cytokines can alter the expression of proteins and RNAs enclosed in NSC-derived exosomes. It was observed that inflammatory cytokine IFN-γ could regulate the exosomal function by altering their miRNA composition, but not affect the secretion of NSC-derived exosomes (Zhang et al., 2020). NSC-derived exosomes have similar active components and functional characteristics to NSCs and have lower immunogenicity and almost zero possibility of malignant transformation (Vogel et al., 2018). Therefore, NSC-derived exosomes may serve as a safer and more effective therapeutic strategy for nervous system diseases.

## Exosome-Mediated Physiological Cross-Talk of Neural Cells

**FIGURE 2:**
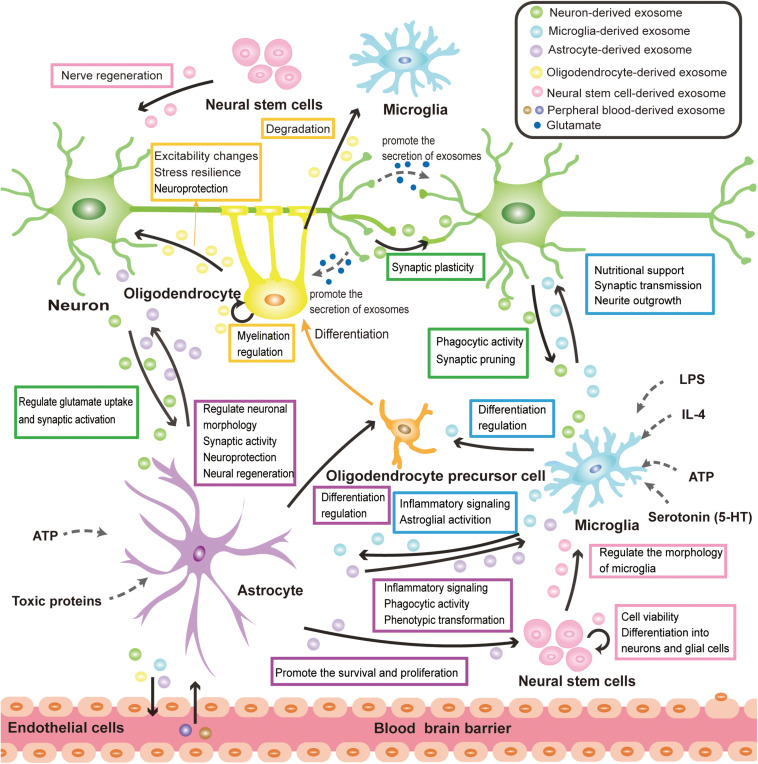
Exosome-mediated physiological cross-talk of neural cells. All major types of neural cells including neurons, microglia, astrocytes, oligodendrocytes, and neural stem cells can secrete and take up exosomes. The communication of exosomes among neural cells can mediate various physiological processes in the CNS. The solid black arrows in the figure represent the direction of exosome transmission, and some of the identified functions for the pathway are labeled on the side. The release of exosomes is mainly modulated by neurotransmitter signals, pathogen components, inflammatory factors, and nutritional stimulation (ATP). These are implied by the dotted arrow in the figure. Exosomes secreted by neural cells can cross the BBB and mediate the communication between CNS and peripheral tissue, which makes exosomes have great potential in disease diagnosis and treatment.

Under physiological conditions, exosomes function as an important information carrier of the CNS, widely involving in the physiological processes of neural cells. This section focuses on the neural cell-derived exosomes and aims to describe their emerging role in the physiological cross-talk between neuron- neuron, neuron-glia, glia-glia, and NSC-neuron/glial cell (Figure 2). At present, the isolation and purification of exosomes still face great challenges. Considering that there are many studies on unclassified EVs of neural cells, besides exosomes, some important researches on EVs will also be mentioned in this section.

### Exosomes in Neuron–Neuron Communication

Increasing evidence indicates that the transmission of exosomes between neurons may be implicated in the modulation of synaptic plasticity. For example, it has been observed that two miRNAs (Let7c and miR21) are present in the exosomes of the supernatant of cultured primary embryonic cortical neurons. Let7c and miR21 may be internalized to the target neurons through exosomes and interact with Toll-like receptor 7 (TLR7), restricting dendritic growth during development in a cell-autonomous manner (Liu et al., 2015). Moreover, the transmembrane protein proline-rich 7 (PRR7) is supposed to be the synapse-removal factor. PRR7 is secreted by neurons in an activity-dependent manner *via* exosomes. Through the fusion of exosomes and neuronal PM, exosomal PRR7 is taken up by neurons, and subsequently inhibits Wnt signaling, activates GSK3β signaling, and induces specific removal of excitatory synapses to maintain the synapse numbers under normal conditions (Lee et al., 2018). Furthermore, postsynaptic retrograde signaling is crucial to the regulation of synaptic growth and plasticity. It was found that the presynaptic membrane can transfer Synaptotagmin 4 (Syt4) to the postsynaptic cell *via* NDEs, thereby activating postsynaptic retrograde signaling and mediating increased neurotransmitter release and activity-dependent presynaptic growth (Korkut et al., 2013). These data demonstrate the significance of ADEs in signal transduction between neurons.

### Exosomes in Neuron-Glia Communication

#### Neuron-Microglia

The neuron-microglia communication is crucial for the growth and development of synapses and functional synaptic transmission. Under physiological conditions, NDEs are putatively involved in the regulation of synaptic pruning by modifying the phagocytic activity of microglia. During neuronal remodeling, exosomes released following neuronal depolarization were demonstrated to up-regulate the levels of complement component 3 (C3) in microglia, thus enhancing the phagocytosis of microglia and facilitating the pruning of inactive synapses (Bahrini et al., 2015). In contrast, microglia may modulate neuronal growth and differentiation and synaptic activity in an exosome-dependent way. A great number of studies highlight the role of MDEs in modulating neuronal energy metabolism and providing nutritional support (Potolicchio et al., 2005; Raffo-Romero et al., 2018). The MDEs have been found containing lactate, which can be oxidized by isoform 1 of lactate dehydrogenase expressed in neurons to produce energy. MDEs may function as energy carriers to meet the energy requirements of increased synaptic activity (Potolicchio et al., 2005). With a model of medicinal leech CNS, Raffo-Romero et al. (2018) found that MDEs exhibit neurotrophic activities and neuro-regenerative effects. These exosomes have been observed to positively influence neurite growth in medicinal leech neurons which may relate to the involvement of nervous Growth/Differentiation Factor (nGDF), a Transforming Growth Factor beta (TGF-β) family member that contained in microglial exosomes (Raffo-Romero et al., 2018). Moreover, microglia-derived MVs have been identified to play a vital role in modulating synaptic transmission (Antonucci et al., 2012; Gabrielli et al., 2015).

#### Neuron-Astrocyte

Neurons can modulate synaptic activity by transferring exosomes to astrocytes. Exosomes containing miR-124a can be transferred from cultured neurons to astrocytes, markedly up-regulating the expression of glutamate transporter-1 (GLT1) in astrocytes, which is believed to modulate extracellular glutamate concentration and synaptic transmission (Morel et al., 2013). The team subsequently demonstrated that neuron-derived miR-124-3p could be internalized by astrocytes through exosomes *in vivo*, and increases GLT1 expression by suppressing the GLT1-inhibiting miRNAs (miR-132 and miR-218) (Men et al., 2019).

Venturini et al. (2019) observed that when neurons and astrocytes were co-cultured, ADEs can be selectively internalized by neurons instead of astrocytes, indicating that exosomes can target neurons, but the specific mechanism has not been elucidated. ADEs can promote neuronal survival and neurite growth. For instance, PrP is an important anti-oxidative stress protein. The non-pathogenic PrP-carrying exosomes secreted by stressed astrocytes can be taken up by neurons and play a neuroprotective role, while PrP-deficient exosomes have no corresponding effect, suggesting that astrocytes potentially enhance the survival rate of neurons upon hypoxic and ischemic stress by releasing exosomes containing PrP (Guitart et al., 2016). Similarly, apolipoprotein D (ApoD), another neuroprotective protein present in ADEs, has been shown to improve neuronal survival under oxidative stress (Pascua-Maestro et al., 2018). Moreover, synapsin I, a synaptic vesicle-associated protein that is implicated in neural development, was found to exist in exosomes secreted by cultured cortical astrocytes and can promote neurite outgrowth by interacting with the neural cell adhesion molecules at the surface of neurons (Wang et al., 2011). ADEs can also impact neurons by regulating synaptic excitability and synaptic morphology. The miRNA-26a-5p carried by ADEs can increase the expression of some neuronal proteins and modulate the dendritic complexity of hippocampal neurons (Luarte et al., 2020). In a recent study, EVs released by astrocytes exposed to ATP have been observed to promote axonal growth, branching, and the firing of neurons (You et al., 2020).

#### Neuron-Oligodendrocyte

As mentioned before, neurons can modulate the secretion of ODEs by releasing neurotransmitters. And ODEs were found to be selectively internalized by neurons and microglia but rarely by oligodendrocytes and astrocytes. The uptake of oligodendrocyte exosomes occurs mainly in the dendrites and axons of neurons and depends on the endocytosis mediated by clathrin and dynamin (Frühbeis et al., 2013). Although the selective mechanism is still unclear, it is plausible to infer that neurons may modulate the protein, nucleic acid, and lipid supply of oligodendrocytes to neurons through this exosome-mediated way, thus affecting the biological function of neurons.

Fröhlich et al. (2014) identified that ODEs can significantly increase the firing rate of cultured neurons, suggesting that oligodendrocytes could enhance the activity of neurons by releasing exosomes. Nevertheless, the influence of ODEs on neuronal activity *in vivo* keeps scarce. Exosomes can also cause the alteration of signal transduction pathways and the gene differential expression in recipient neurons (Fröhlich et al., 2014). In another recent study, ODEs were showed to reduce the pausing time of vesicles and increase anterograde vesicle movement, thus promoting rapid transport of brain-derived neurotrophic factor (BDNF)-contained vesicles in the axons which are important to the maintenance of axonal homeostasis (Frühbeis et al., 2020). Moreover, ODEs have been shown to improve neuronal survival by delivering protective substances and promoting axon transport. For instance, under oxygen-glucose deprivation (OGD) conditions, the neuroprotective effect of ODEs is directly related to their carried enzymes (superoxide dismutase and catalase), which are thought to protect cells against oxidative stress and encourage the survival of neurons (Fröhlich et al., 2014). In conclusion, these studies suggest that the transmission of exosomes from oligodendrocytes to neurons may serve as a glial support mechanism, providing nutritional metabolic support for neurons to meet their activity requirements, and mediating the neuronal protection under stress conditions, thus promoting the maintenance of neuronal homeostasis.

### Exosomes in Glia–Glia Communication

As the innate immune cells of the CNS, the communication between microglia and astrocyte is crucial for neuroinflammation modulation. Astrocyte-derived ATP can promote the EVs including exosomes and MVs shedding from microglia (Bianco et al., 2005; Takenouchi et al., 2015). Released MDEs and MVs can increase the expression of cytokines such as IL-1, IL-6, TNF-α in astrocytes, and promote astroglial activation (Drago et al., 2017). Activated astrocytes are extensively involved in the regulation of CNS inflammatory response (Colombo and Farina, 2016). However, how these MDEs and MVs regulate the function of astrocytes remains unclear. Astrocytes can also modulate the microglia-mediated inflammatory response through exosomes. After systemic immune activation, the expression of histocompatibility complex class I (MHC I) in astrocytes of mice medial prefrontal cortex increased. MHC I and its encoding gene H-2D may be transported to microglia cells and neurons through ADEs to activate microglia and reduce the density of the dendritic spine, thereby mediating nerve injury in mice. And these impairments were significantly alleviated with the treatment of an exosome synthesis inhibitor GW4869 (Sobue et al., 2018). Moreover, a recent study revealed that ADEs enabled to alleviate microglia-mediated neuroinflammation by promoting the transformation of microglia to the M2 phenotype. This process depended on the suppression of the NF-κB signaling pathway in microglia by exosomal miR-873a-5p (Long et al., 2020). Besides, the EVs secreted by astrocytes can also modulate the phagocytic activity of microglia. Long intergenic noncoding RNA (lincRNA)-Cox2 plays a critical role in the modulation of immune-related genes in microglia. EVs produced by morphine-stimulated astrocytes were found to be taken up by microglia and activate the TLR7-nuclear factor κB (NF-κB)-lincRNA-Cox2 signaling pathway, thus impairing microglial phagocytosis, which will affect the ability of microglia to phagocytize and remove pathogens or cell debris (Hu et al., 2018).

Fitzner et al. (2011) discovered that ODEs could be selectively internalized by microglia through macropinocytosis without causing the immune activation of microglia, suggesting that microglia may mediate the clearance of oligodendrocyte membrane in an immunologically “silent” manner through the uptake of ODEs. Microglia are involved in myelin damage and regeneration by regulating the function of oligodendrocyte precursor cells (OPCs) (Traiffort et al., 2020). Lombardi et al. (2019) have shown the function of microglia EVs in this process. Under different stimulations, activated microglia can secrete EVs with pro-inflammatory or pro-regenerative activities, which respectively inhibit or promote the differentiation of OPCs to myelin-forming cells (oligodendrocytes) (Lombardi et al., 2019). Furthermore, when cultured OPCs are exposed to inflammatory EVs released by microglia, maturation of OPCs is hindered only when astrocytes are present, suggesting that microglial EVs may suppress the remyelination by converting astrocytes into myelin sheath-injured cells. Additionally, although the role of EVs in regulating OPCs maturation is unclear, this study indicates that sphingosine 1 phosphate on the surface of EVs can stimulate OPCs migration. Therefore, the lipids of EVs may serve as a critical part in the modulation of remyelination (Lombardi et al., 2019).

Astrocytes are essential for oligodendrocyte survival and axonal myelin formation of axons and are also a source of nutritional support for maintaining myelin integrity (Nutma et al., 2020). Nevertheless, to our knowledge, few reports reveal the role of exosomes in astrocyte-to-oligodendrocyte communication. Astrocytes can also secrete a set of cytokines to promote the survival, proliferation, and maturation of OPCs (Nutma et al., 2020). A recent study found that EVs secreted by young astrocytes (cultured *in vitro* ≤4 weeks) could promote the differentiation of OPCs into oligodendrocytes, while this effect was reduced with EVs from old astrocytes (cultured *in vitro* ≥16 weeks). Further proteomic analysis showed that the proteins of EVs released by astrocytes also changed with the increase of culture time. This study supports the idea that astrocytes can provide support for oligodendrocyte differentiation through exosomes, and aging may reduce their ability to provide homeostasis support (Willis et al., 2020).

Oligodendrocytes can negatively regulate their differentiation and myelin formation in an autocrine manner. Exosomes isolated from rat primary oligodendrocytes were found to influence actomyosin contractility through activating the Rho-ROCK (Rho-associated kinase)-myosin signaling axis on the recipient oligodendrocytes, thus avoiding cell surface expansion and inhibiting the oligodendroglial differentiation and myelin formation. Interestingly, this study also showed that the secretion of self-inhibiting exosomes was significantly down-regulated by incubating oligodendrocytes with conditioned neuronal medium, indicating that neurons possibly participate in the regulation of oligodendrocyte differentiation and myelin formation by controlling the release of ODEs, which may contribute to the maintenance of brain homeostasis (Bakhti et al., 2011).

### Exosomes in NSC-Neuron/Glial Cell Communication

Neural stem/progenitor cells are involved in regulating the physiological activities of recipient cells through exosomes. EVs from both mouse and human NSCs contain asparaginase-like protein 1 (Asrgl1), which has L-asparaginase catalytic activity and can increase the level of aspartate in the extracellular microenvironment, thereby regulating the energy metabolism of surrounding cells (Iraci et al., 2017). Proinflammatory cytokine (Th1-like cells) induced NSCs can transfer IFN-γ to the target cells *via* exosomes, activating the STAT1 signaling pathway and mediating the activation of pro-inflammatory signaling in target cells (Cossetti et al., 2014). Stevanato et al. (2016) found that the exosomes secreted by human NSCs were significantly enriched in miR-1246, which has been shown to play an important role in regulating cell growth and apoptosis.

A few studies have preliminarily revealed the role of exosomes in the communication between NSCs and other neural cells. It was demonstrated that mild hypoxia can induce NSCs to secrete exosomes containing miR-210, which can be taken up by recipient NSCs and increase cell viability, whereas severe hypoxia or serious doses of miR-210 may reduce the viability of NSCs (Zhao et al., 2020). Another study has shown that ADEs can also promote the survival and proliferation of NSCs (Zhou et al., 2020). Moreover, NSC-derived exosomes play an important role in neurogenesis by mediating the differentiation of NSCs. A recent study showed that exosomes secreted by NSCs can promote the differentiation of recipient NSCs and subsequent maturation of neurons and glial cells through the miR-9-Hes1 pathway (Yuan et al., 2021). Stronati et al. (2019) also found that exosomes produced by differentiated NSCs may promote the differentiation of NSCs in a dose-dependent manner. MiR-21a was observed to be highly enriched in NSC-derived exosomes and could promote the differentiation of NSCs into neurons (Ma et al., 2019). Furthermore, several studies have highlighted the role of NSC-derived exosomes in regulating the physiological activity of neurons and microglia. NSC-derived exosomes have the effect of promoting nerve regeneration. An *in vitro* study has shown that exosomes released from NSCs can promote neuronal axonal elongation and neural network reconstruction under both normal and hypoxia-reperfusion injury conditions (Liu Q. et al., 2020). Morton et al. (2018) demonstrated that exosomes released by NSCs of neonatal SVZ can be selectively ingested by microglia. These exosomes contain miR-9, let-7, miR-26, and miR-181, which can regulate the morphology of microglia and increase the secretion of cytokines, thereby mediating negative feedback and reduce the proliferation of NSCs (Morton et al., 2018). Collectively, these results implied that NSC-derived exosomes have a potential role in neurodevelopment and homeostasis maintenance of the CNS.

## Potential Role of Neural Cell-Derived Exosomes in the Pathology and Diagnosis of Neurodegenerative Diseases

Neural cell-derived exosomes seem to be involved in neurodegenerative diseases mainly through the transmission of toxic proteins and neuroinflammation. The role of miRNAs carried by exosomes in neurodegenerative diseases has also attracted increasing attention. Moreover, besides mediating nerve damage, neural cell-derived exosomes also show a protective effect in neurodegenerative diseases, and the dual effects of exosomes may depend on different extracellular environments and cell states. Exosomes secreted by neural cells can cross the BBB and be detected in body fluids. Like the fingerprint of neural cells, exosomes can well reflect the physiological and pathological state of parental cells, and carry proteins, RNA, lipids, and other bioactive substances to the periphery, making it possible for the early recognition of neurodegenerative diseases.

### Alzheimer’s Disease

Alzheimer’s disease (AD) is characterized by the deposition of the toxic beta-amyloid (Aβ) protein in the extracellular matrix between neurons, and the formation of neurofibrillary tangle due to the abnormal phosphorylation of tau protein, which could further induce neuroinflammation, oxidative stress, neurodegeneration and even neuronal apoptosis (Yin et al., 2020). Many recent studies have indicated that neural cell-derived exosomes have multiple roles in the occurrence, development, and early diagnosis of AD (Table 3).

**TABLE 3:** The role of neural cell-derived exosomes in AD pathology.

	Function	Exosome-producing cells	Stimulation	Cargo	Mechanism	References
Neuron	Propagation of toxic proteins	Neurons from AD brain	–	oAβ	Mediating the propagation of oAβ between neurons and causing cytotoxicity.	Sardar Sinha et al., 2018
		Rat embryos cortical neurons and neuroblastoma N2a cells	–	Tau protein	Mediating the trans-synaptic transmission of tau protein.	Wang et al., 2017
	Clearance of toxic proteins	Primary cortical neurons from mouse and neuroblastoma N2a cells	–	–	Promoting the conformational transformation of Aβ to non-toxic amyloid fibrils and delivery to microglia for degradation.	Yuyama et al., 2012
		Primary cortical neurons from mouse brains	–	Glycosphingolipid	Capturing Aβ and decreasing the deposition of Aβ and amyloid.	Yuyama et al., 2015
		Primary cortical neurons from mouse and neuroblastoma N2a cells	Vps34 kinase inhibitor VPS34IN1 (inducing the lysosomal dysfunction of neurons)	Undigested lysosomal substrates (amyloid precursor protein C-terminal fragments, specific sphingolipids, etc.)	Mediating the clearance of toxic proteins to maintain neuronal homeostasis.	Miranda et al., 2018
Microglia	Propagation of toxic proteins	Primary cultured murine microglia	Pre-aggregated human tau 1–441 protein, ATP and LPS	Tau protein	Mediating the propagation of tau protein to neurons.	Asai et al., 2015
	Transmission of neuroinflammation	Primary mouse microglia	LPS, IL-4, IL-10	Up-regulated pro-inflammatory miRNAs and down-regulated anti-inflammatory miRNAs	Facilitating the transmission of neuroinflammation.	Gao et al., 2019
Astrocyte	Propagation of toxic proteins	Untransformed cortical adult human astrocytes	Aβ_25__–__35_	Tau and p-Tau	The spreading of tau in the brain.	Chiarini et al., 2017
	Transmission of neuroinflammation	ADEs from plasma samples of AD patients	–	IL-6, TNF-α, IL-1β, Complement proteins	Mediating neuroinflammation and neuronal damage in late AD.	Goetzl et al., 2018b
	Apoptosis of astrocytes	Cortical primary astrocytes from pups	Amyloid protein	Prostate apoptosis response 4 (PAR-4), ceramide	Promoting neuronal apoptosis by activating caspase 3.	Wang et al., 2012

Mounting evidence suggests that exosomes are capable of mediating the propagation of toxic proteins, both Aβ and hyperphosphorylated tau (p-tau) among neural cells, and further inducing apoptosis and promote the development of AD. For example, it has been confirmed that NDEs are implicated in the transmission of Aβ oligomers (oAβ) between neurons, and blocking the formation and secretion of NDEs can suppress the spread of oAβ (Sardar Sinha et al., 2018). Trans-synaptic transmission of tau may also be mediated by NDEs, and the release of NDEs containing tau is regulated by synaptic activity (Wang et al., 2017). Moreover, tau protein and Aβ were found existing in AD transgenic mice serum exosomes which possibly released from astrocytes, suggesting that ADEs may be important to the pathological transmission of AD (Rosas-Hernandez et al., 2019). Chiarini et al. (2017) found that exposure to Aβ can significantly increase the expression of tau and p-tau in astrocytes and promoted the release of ADEs carrying tau and p-tau, thereby promoting the spread of tau protein in the brain. Furthermore, MDEs also participate in the propagation of toxic proteins. Microglia can deliver the internalized tau to neurons through exosomes, promoting the pathological spread of tau. The transmission of tau *via* MDEs was proved to be more efficient than the naked form *in vivo*, and the microglial depletion and inhibition of MDEs synthesis can significantly reduce tau proliferation both *in vivo* and *in vitro* (Asai et al., 2015). Taken together, the described researches document that exosomes may be a key mediator in the propagation of toxic proteins and conduce to the neuronal damage in AD.

Neuroinflammation is viewed as a key mechanism of AD, and recent studies highlighted the role of MDEs and ADEs in AD-related neuroinflammation. Glutaminase C (GAC) was found to be abnormally elevated in the brain tissues of early AD mice. With the stimulation of pro-inflammatory factors (LPS, IL-4, and IL-10), GAC can be specifically up-regulated in microglia, thus inducing cell activation, exosome secretion, as well as the content modification of exosomes. The released MDEs contain up-regulated pro-inflammatory miRNAs and down-regulated anti-inflammatory miRNAs, thereby facilitating the transmission of neuroinflammation in AD (Gao et al., 2019). A1-type astrocytes that are up-regulated in AD patients have been reported to secrete exosomes with high levels of complement proteins (CPs), which may contribute to neuronal injury in the late inflammatory phase of AD (Goetzl et al., 2018b).

Besides the propagation of toxic proteins and neuroinflammation, neural cell-derived exosomes can also contribute to the neurodegeneration of AD through other pathways. It has been demonstrated that amyloid peptide-treated astrocytes can release exosomes containing proapoptotic proteins. These ADEs accumulated in the medium and bound to other astrocytes after reaching the proapoptotic concentration, thus promoting apoptosis by activating caspase 3. This study revealed a new mechanism of exosome-mediated astrocytes apoptosis under pathological conditions (Wang et al., 2012).

However, some other evidence indicated that the release of NDEs can also serve as a neuroprotective role to maintain neuronal homeostasis by promoting the clearance of toxic proteins. It was discovered that NDEs could promote the conformational transition of Aβ in the extracellular matrix and form non-toxic amyloid fibrils on the exosomal surface. Following the internalization of NDEs, the Aβ amyloid fibrils on the exosomal surface were internalized by microglia and further transported to lysosomes for degradation. Sphingomyelin synthase 2 (SMS2) has been shown to up-regulate the secretion of NDEs during this process (Yuyama et al., 2012). Moreover, with abundant glycosphingolipids, NDEs have a good ability to capture Aβ. The infusion of NDEs could reduce the deposition of Aβ in the brains of amyloid precursor protein (APP) transgenic mice, suggesting the crucial role of NDEs in Aβ clearance (Yuyama et al., 2015). Additionally, AD is believed to be closely related to the dysfunction of neuronal endolysosome mediated by phosphatidylinositol-3-phosphate (PI3P) deficiency. Neurons with endolysosomal dysfunction can compensatively secrete exosomes containing APP C-terminal fragments (APP-CTFs), thereby mediating the clearance of toxic proteins to maintain neuronal homeostasis (Miranda et al., 2018).

Multiple studies have revealed that neural-derived exosomes detected from peripheral body fluids carry specific proteins and genetic cargos, enabling to realize the early identification of AD at the preclinical stage. Regarding the exosomal protein biomarker of AD, the initial studies mainly focused on Aβ and Tau. It has been demonstrated that the levels of Aβ1–42, p-Tau 181, and p-Tau 396 in blood NDEs significantly increased in AD than healthy individuals and can predict the progression of AD 10 years before the clinical stage (Fiandaca et al., 2015; Winston et al., 2016). These pathogenic proteins in NDEs have also been found to be closely associated with the severity of clinical symptoms and are useful biomarkers for monitoring AD progression (Nam et al., 2020). In particular, it was confirmed that Aβ42, T-Tau, and P-T181-Tau in NDEs had the same ability to diagnose AD as those in CSF, thus providing a more convenient and less invasive method for the definite diagnosis of AD (Jia et al., 2019). Moreover, other protein biomarkers associated with synaptic pathology, lysosomal dysfunction, and decreased glucose metabolism or insulin resistance have also been discovered in NDEs of AD patients. These protein biomarkers have been observed to have a significant difference between AD and normal exosomes, and these changes are already present at the early stages of the disease (Goetzl et al., 2018a; Soares Martins et al., 2021). In addition, the possibility of proteins contained in ADEs as biomarkers has also been investigated. Studies by Goetzl et al. showed that the ADEs of AD patients load a variety of differentially expressed proteins, including β-site APP-cleaving enzyme 1 (BACE-1), soluble amyloid precursor protein (sAPP) β and glial-derived neurotrophic factor (GDNF). However, whether they can be used as biomarkers for early recognition of AD needs further study (Goetzl et al., 2016). The levels of inflammatory cytokines and CPs in plasma ADEs of AD patients were significantly higher than those in normal controls, which may be helpful for the early detection of AD (Goetzl et al., 2018b).

Gene cargo carried by exosomes also serves as biomarkers for early recognition of AD, in which exosomal miRNAs have been extensively studied and well described in some reviews (Manna et al., 2020; Soares Martins et al., 2021). At present, most studies on exosomal miRNA biomarkers have been carried out in total exosomes without identification of their origin. It has been described that miR-212 and miR-132 are decreased in plasma NDEs from AD patients, but their potential as AD biomarkers still should be further investigated (Cha et al., 2019). Future identification of the cellular origin of these exosomes will be necessary to understand the targeting mechanisms of miRNAs and to achieve a more accurate diagnosis.

### Parkinson’s Disease

The hallmarks of Parkinson’s disease (PD) pathology are the degeneration of dopamine neurons in the substantia nigra pars compacta and the progressive accumulation of α-synuclein (α-syn) and the formation of oligomers in neurons (Marchetti et al., 2020). Neural cell-derived exosomes are considered as important mediators for the transmission of PD pathology and may be pivotal in the occurrence and progression of PD (Table 4).

**TABLE 4:** The role of neural cell-derived exosomes in PD pathology.

	Function	Exosome-producing cells	Stimulation	Cargo	Mechanism	References
Neuron	Propagation of toxic proteins	Differentiated SH-SY5Y cell line	Over-expression of wild-type α-syn	α-syn	Propagation of α-syn and triggering neuronal apoptosis.	Emmanouilidou et al., 2010; Alvarez-Erviti et al., 2011
	Protein aggregation and inflammation	MN9D cell model	Environmental neurotoxicant manganese	miR-210-5p, miR128-1-5, miR-325-5p, miR-16-5p. etc.	Carrying miRNA involving in protein aggregation, autophagy, inflammation, and hypoxia and aggravating the degeneration of PD.	Harischandra et al., 2018
Microglia	Propagation of toxic proteins	BV-2 microglial cell line	Plasma exosomes from PD patients/human α-synuclein preformed fibrils	α-syn	α-syn propagation and the aggregation of α-syn in recipient neurons.	Xia et al., 2019; Guo et al., 2020
	Transmission of neuroinflammation	BV-2 microglial cell line	α-syn	MHC II, TNF-α	Inducing neuroinflammatory response and neuronal apoptosis.	Chang et al., 2013
Astrocyte	Neuronal protection	Primary mouse astrocyte	–	miR-200A-3p	Inhibiting apoptotic signaling pathway and reducing MPP^+^-induced apoptosis of hippocampal neurons.	Shakespear et al., 2020

It is increasing evidence that the aggregation and propagation of α-syn are at least partially dependent on exosomes. A recent study reported that although α-syn can be released directly by neurons, pathogenic forms of α-syn may be more commonly secreted through exosomes and other EVs. EVs carrying α-syn may be more prone to internalized by the recipient cells, highlighting the unique advantage of EVs in the transmission of toxic proteins (Gustafsson et al., 2018). Neurons affected by toxic proteins can transfer α-syn to healthy neurons through NDEs, thus promoting the pathological transmission of PD (Alvarez-Erviti et al., 2011). Emmanouilidou et al. (2010) found that differentiated SH-SY5Y cells are capable of secreting exosomes containing α-syn in a calcium-dependent manner, and the released exosomes were observed to induce neuronal death. The potential role of microglia in the pathologic spreading of α-syn is revealed by Xia et al. (2019) and Guo et al. (2020) in their recent work. They respectively demonstrated that exposure to plasma exosomes from PD patients or human α-synuclein preformed fibrils can reduce the clearance efficiency of microglia and induce the release of α-syn-enriched exosomes, thereby facilitating α-syn propagation and the aggregation of α-syn in the target neurons (Xia et al., 2019; Guo et al., 2020).

Exosomes in the CNS also participated in microglial activation and neuroinflammatory, which aggravate the degeneration of dopamine neurons. It has been found that α-syn can stimulate microglia to produce inflammatory exosomes rich in MHC II and TNF-α, thereby inducing neuroinflammatory response and apoptosis of rat cortical neurons (Chang et al., 2013). Intriguingly, aging-related microglial dysfunction seems to be closely related to the pathogenesis of PD. Bliederhaeuser et al. (2016) found that compared with young mice, microglia in adult mice showed deficits in exosome-associated α-syn uptake and phagocytosis, which facilitates the diffusion of toxic proteins between neurons and exacerbates degenerative changes. Additionally, aging microglia can also release more exosomes containing TNF-α than young microglia, further damaging neurons by aggravating the inflammatory response. This observation illustrated that age is an important factor affecting the spreading of α-syn and neuroinflammation *via* exosomes (Bliederhaeuser et al., 2016).

The effect of exosomes on PD pathology may be mediated by specific miRNAs. Manganese exposure is thought to promote the progression of PD (Kwakye et al., 2015). Notably, it was found that MN9D cells (a dopaminergic cell model of PD) exposed to manganese upregulated the Rab27a, which was been demonstrate to promote the exosomal release. The released NDEs are rich in miRNAs associated with protein aggregation, autophagy, inflammation, and hypoxia, and may facilitate the progression of PD (Harischandra et al., 2018).

Exosomes from neural cells are not always harmful, and they can also show neuroprotective effects in AD under the influence of the extracellular environment and physiological state of cells. As an example, 1-methyl-4-phenylpyridinium (MPP^+^), a neurotoxin that could induce cell death, is widely used in models of PD both *in vivo* and *in vitro*. Exosomes released from normal astrocytes were found to significantly reduce MPP^+^-induced apoptosis of hippocampal neurons. This may be because miR-200A-3p in the ADEs can down-regulate the mitogen-activated protein kinase kinase 4 (MKK4) expression in MPP^+^-induced neurons and further inhibit the activation of c-jun n-terminal kinase (JNK), which is essential in the apoptotic signaling pathway (Shakespear et al., 2020).

In the last few years, mounting evidence points to a role for exosomes as biomarkers to assist in the clinical diagnosis and prognosis of PD. Multiple studies have examined the potential of exosome-carried α-syn and other proteins as biomarkers in different biological fluids of PD (Yu et al., 2020). Indeed, these exosomal protein biomarkers present in peripheral body fluids may be contaminated as they can also be secreted by peripheral tissues. Therefore, it is indispensable to identify the source of exosomes. Although some studies have analyzed the level of neuron-derived exosomal α-syn in the peripheral blood of PD patients, the results were inconsistent. Two studies on PD individuals’ plasma NDEs showed that the α-syn from NDEs was significantly higher than that of normal subjects, and the levels of α-syn increased with the progression of PD (Zhao et al., 2018; Niu et al., 2020). Jiang et al. also found similar results in serum NDEs (Jiang et al., 2020). In the study of Si et al. (2019), levels of neuron-derived exosomal α-syn in serum of PD patients were observed to be lower than those in controls. The lack of standardized protocol for identifying NDEs may be responsible for this difference. Studies of exosomal miRNAs from PD patients’ serum or CSF have demonstrated that the enrichment or deficiency of specific miRNAs may contribute to the diagnosis of PD (Gui et al., 2015; Cao et al., 2017; Wang and Zhang, 2020). However, it is challenging to identify the origin of these exosomes carrying functional miRNAs. Considering the prominent role of exosomes secreted by neural cells in PD pathology, it is to distinguish the neural origin of these exosomes in future biomarker studies.

### Amyotrophic Lateral Sclerosis

Regarding amyotrophic lateral sclerosis (ALS), mutations of copper-zinc superoxide dismutase (SOD1) are one of the important causes of inherited ALS. Exosomes from neural cells seem to serve as an important part of the pathological development of ALS. Basso et al. (2013) found that exosomes secreted by mutant copper-zinc superoxide dismutase (SOD1) overexpressed astrocytes were shown to transport mutant SOD1 to cultured spinal neurons and selectively induce the injury of motor neurons. Neuroinflammation is one of the main pathogenic mechanisms of ALS, it is described that IL-6 in ADEs is up-regulated in sporadic ALS patients, and the disease progression rate of ALS is positively correlated with the level of IL-6 in ADEs. It is speculated that ADEs aggravate the ALS process by inducing inflammatory responses (Chen et al., 2019). Moreover, in a study by Pinto et al. (2017), exosomes secreted by NSC-34 motor neuron-like cells transfected with mutant SOD1 (G93A) were found containing miR-124. The miR-124-enriched NDEs were preferentially internalized by microglia, modulating the cell phenotype and triggering the microglial dysfunction by inducing the loss of phagocytic ability and the production of multiple inflammatory cytokines and MHC-II (Pinto et al., 2017). The damaged microglia have a lower ability to eliminate harmful substances and maintain neural homeostasis, as well as mediated the enhancement of neuroinflammation, thus further promoting the degeneration of motor neurons (Pinto et al., 2017).

Several researches point to a role for exosome-carried proteins as biomarkers of ALS (Chen P.C. et al., 2020; Hayashi et al., 2020). Similarly, studies of these exosomal biomarkers are based on total exosomes in biological fluids, few studies have focused on protein biomarkers from neuronal and glial cell-derived exosomes. Several studies have analyzed miRNAs carried by NDEs aiming to screen for potential biomarkers. Yelick et al. (2020) found that the elevation of exosome containing miR-124-3p from spinal neurons of patients is positively correlated with the severity of ALS, highlighting the potential role of exosome miR-124-3p as an indicator of ALS disease stage. Plasma NDEs-derived miRNAs were also identified by Banack et al. (2020) in the ALS group. In contrast to the controls, eight miRNA sequences were observed to be differentially expressed in NDEs of ALS, which may contribute to the early diagnosis of ALS (Banack et al., 2020).

Collectively, these studies demonstrated that exosomes from neurons, microglia, and astrocyte are involved in the occurrence and development of neurodegenerative diseases and this role is twofold, involving both nerve damage and neuroprotection. In the future, how to distinguish the beneficial or harmful exosomes under different physiological and pathological conditions is crucial for the development of new exosome therapies for neurodegenerative diseases. Oligodendrocytes are responsible for CNS myelination and provide nutritional support to axons and neurons. Dysfunction of oligodendrocytes and myelin may increase the vulnerability of neuronal degeneration (Ettle et al., 2016). However, the role of ODEs in neurodegenerative diseases still needs further investigation. Exosomes are also an important tool for the early non-invasive diagnosis of neurodegenerative diseases. However, most current studies are based on total exosomes in peripheral body fluids, and the role of neural cell-derived exosomes has not been fully explored. Although some studies have reported the exosomal origin of biomarkers, the current markers used to identify neural cell-derived exosomes seem to be unspecific. For instance, L1CAM, which is widely used in the identification of NDEs, may also be expressed by the kidney and adipose tissue. Further research on accurate identification of specific cell-derived exosomes is conducive to accurate diagnosis and prognosis prediction of neurodegenerative diseases.

## Possible Therapeutic Strategies Based on Exosomes

Applications of exosomes in the therapy of neurodegenerative diseases are embodied in three aspects: one is as a promising target for disease therapy, the second is the use of stem cell-derived exosomes, and the third is to design exosomes as drug delivery vehicles.

Exosomes secreted by neural cells perform an increasingly vital role in spreading toxic proteins and mediating neuroinflammation, contributing to the occurrence and development of neurodegenerative diseases. Therefore, blocking the formation of neural exosomes or their internalization by target cells may be emerging targets for the therapy of neurodegenerative diseases. As an example, a recent study has shown that inhibiting P2RX7 can suppress ATP-induced secretion of tau-containing MDEs, thus significantly decreasing the accumulation of tau protein and improving the working memory of AD mice. This study suggests that P2RX7 may be a novel therapeutic target of AD by inhibiting exosome secretion (Ruan et al., 2020). However, studies on therapeutic interventions targeting exosome-related molecular are still lacking. Further studies on the mechanism of neural exosomal occurrence, content modification, secretion, and targeting recipient cells are beneficial to the identification of new therapeutic targets of neurodegenerative diseases. In the future, the development of drugs inhibiting the pathological activities of exosomes may be an exciting direction. Nevertheless, as mentioned above, neural cell-derived exosomes also serve a beneficial role in neurodegenerative diseases. In this sense, it seems that inhibition of exosomal secretion for therapy should be treated with caution. In conclusion, further understanding the effects of neural exosomes in degenerative diseases, as well as clarifying the conditions that mediate beneficial and harmful exosome production in the context of diseases, is critical to the development of effective neural exosome-based therapies.

Most current research on exosome-mediated therapy has focused on the use of exosomes from stem cells. Stem cell-derived exosomes have similar active components and functional characteristics to stem cells and have lower immunogenicity and almost zero possibility of malignant transformation. Therefore, stem cell exosomes may serve as a safer and more effective therapeutic strategy for neurodegenerative diseases. A great number of studies highlighted the great potential of stem cell exosomes in the therapy of neurodegenerative diseases. Human mesenchymal stem cells (MSCs) secreted exosomes contain a range of therapeutic nucleic acids, proteins, and lipids that have been widely believed to reduce neuroinflammation and improve clinical symptoms of AD and PD (Chen H.X. et al., 2020; Nakano et al., 2020). Exosomes released by adipose stem cells have also been reported to carry a variety of proteins that serve a neuroprotective role in ALS by regulating apoptosis (Bonafede et al., 2019). Furthermore, some studies have revealed the role of neural stem cell-derived exosomes in the treatment of degenerative diseases. It has been identified that exosomes released by hippocampal neural stem cells contained specific miRNAs, which can enhance synaptic resistance to amyloid beta oligomers (Aβo) and rescue Aβo-induced long-term potentiation inhibition and memory deficits (Micci et al., 2019). AD is associated with the deficiency of BBB. Using an *in vitro* BBB model of AD, Liu Y. et al. (2020) showed that BBB disruption induced by AD could be repaired by NSC-derived exosomes.

Especially, exosomes are potential drug delivery vehicles. Dependent on transferrin-transferrin receptor interactions, exosomes in the blood appears to have good brain targeting capabilities that allow drugs to cross the BBB and perform their pharmacological effects more efficiently (Qu et al., 2018). Wang et al. (2019) first explored the potential of exosomes as therapeutic vectors for AD. When exosomes containing curcumin were intravenously injected into AD rats, curcumin carried by exosomes was observed to cross the BBB and highly accumulated in the brain of AD rats, and further, alleviate symptoms of AD by inhibiting hyperphosphorylation of tau. Exosomes significantly improved the solubility and bioavailability of curcumin (Wang et al., 2019). Dopamine replacement therapy can effectively improve the motor symptoms of PD patients. It has been described demonstrated that dopamine-loaded exosomes can effectively improve the symptoms of PD mice, and behave better in therapeutic efficacy and lower systemic toxicity than free dopamine (Qu et al., 2018). These studies indicated that exosomes can be a novel drug delivery tool for the targeted therapy of neurodegenerative diseases.

Consequently, it is becoming increasingly evident that exosomes have great capacity in the therapy of neurodegenerative diseases. However, there are also many challenges. First, exosomes injected peripherally may be cleared by immune cells. How to safely and efficiently deliver designed exosomes to specific targets in CNS still needs further research. Secondly, exosomes carrying specific therapeutic active molecules may elicit immune responses in patients. Furthermore, most of the current research comes from *in vitro* and animal experiments, with a lack of clinical trials. These issues need to be further overcome in future studies. In addition, further research is needed to deepen our understanding of neural exosomes in neurodegenerative diseases and clarify whether they have a potential advantage in designing new therapeutic strategies.

## Conclusion and Future Perspective

In the last decade, exosomes have attracted extensive attention as important carriers of intercellular information exchange. This review provides preliminary evidence for the function of neural exosomes in CNS intercellular communication in both health and neurodegenerative diseases. However, the field of neural cell-derived exosomes in the CNS is still in its infancy and faces many problems and challenges. So far, most researches on the neural exosomes are performed in cell culture (*in vitro*) or by injecting or adding exosomes obtained from cell culture into animal models. Whether these studies can reflect the characteristics and function of exosomes *in vivo* needs to be further clarified. More animal and clinical researches are needed to verify the neural exosome-mediated physiological and pathological processes *in vivo*. Moreover, because of the overlap of EVs in size, composition, and biochemical markers, the isolation and purification of exosomes remain a challenge, so some of the results discussed in this article may reflect the function of exosomes mixed with other EVs. The development of techniques to accurately isolate these vesicle subtypes will help us better understand the unique properties of exosomes in neural cell communication. In addition, to fully understand the role of exosomes in the intercellular and diseases of the CNS, more basic work is needed to elucidate the biological characteristics of neural cell-derived exosomes, such as the mechanisms of biogenesis and content sorting, how to target recipient cells (whether the targeting is specific and the specific mechanisms), and how to participate in complex biological processes in recipient cells. Given the dual effect of exosomes in the CNS, recognition of conditions that induce beneficial or harmful exosome secretion, and research of related intracellular mechanisms are beneficial for disease intervention from the exosome level.

In summary, as a novel information transfer carrier, neural cell-derived exosomes perform an increasingly vital role in the intercellular communication of the CNS, mediating the regulation of physiological activities, as well as the damaged or protective effect under neurodegenerative diseases. The study of neural exosomes in the CNS may contribute to the development of new diagnostic and therapeutic strategies for neurodegenerative diseases.

## Author Contributions

YX, SL, and LH provided the theme and ideas of this manuscript. LH, XD, and XL consulted the relevant literature. LH and XD wrote the manuscript. LH, XD, SL, XL, and YX revised the manuscript. All authors approved the final manuscript and agreed to be accountable for all aspects of the work.

## Conflict of Interest

The authors declare that the research was conducted in the absence of any commercial or financial relationships that could be construed as a potential conflict of interest.

## Publisher’s Note

All claims expressed in this article are solely those of the authors and do not necessarily represent those of their affiliated organizations, or those of the publisher, the editors and the reviewers. Any product that may be evaluated in this article, or claim that may be made by its manufacturer, is not guaranteed or endorsed by the publisher.
